# Preparation and Strength Formation Mechanism of Calcined Oyster Shell, Red Mud, Slag, and Iron Tailing Composite Cemented Paste Backfill

**DOI:** 10.3390/ma15062199

**Published:** 2022-03-16

**Authors:** Hongxu Lu, Qi Sun

**Affiliations:** 1 School of Civil Engineering, Liaoning Technical University, Fuxin 123000, China; luhongxu2022@163.com; 2 College of Architecture and Transportation, Liaoning Technical University, Fuxin 123000, China

**Keywords:** iron tailing, calcined oyster shell, red mud, CCD, quantum genetic algorithm, strength formation mechanism

## Abstract

The use of bulk solid-waste iron tailing (IOT), red mud (RM), and oyster shells to prepare cemented paste backfill (CPB) can effectively solve the ecological problems caused by industrial solid waste storage and improve the utilization rate of such materials. In this study, a new type of CPB was prepared by partially replacing slag with RM, with calcined oyster shell (COS) as the alkaline activator and IOT as aggregate. The central composite design (CCD) method was used to design experiments to predict the effects of the COS dosage, RM substitution rate, solid mass, and aggregate–binder ratio using 28-dUCS, slump, and the cost of CPB. In this way, a regression model was established. The quantum genetic algorithm (QGA) was used to optimize the regression model, and X-ray diffraction (XRD), Fourier transform infrared (FTIR), scanning electron microscope (SEM), and energy dispersive spectroscopy (EDS) microscopic tests are performed on CPB samples of different ages with the optimal mix ratio. The results showed that COS is a highly active alkaline substance that provides an alkaline environment for polymerization reactions. In the alkaline medium, the hematite and goethite in RM and quartz in IOT gradually dissolved and participated in the process of polymerization. The main polymerization products of the CPB samples are calcium–silicate–hydrogel (C–S–H), calcium–aluminosilicate–hydrogel (C–A–S–H), and aluminosilicate crystals such as quartz, albite, and foshagite. These products are intertwined and filled in the internal pores of the CPB, enabling the pore contents to decrease and the interiors of the CPB samples to gradually connect into a whole. In this way, the compressive strength is increased.

## 1. Introduction

IOT, RM, and oyster shells, as bulk solid wastes, have a serious negative impact on the ecological environment. As of 2013, China’s IOT output was 5 billion tons, but the utilization rate was less than 20%, and China was the fourth largest alumina producer in the world, emitting tens of millions of tons of RM every year [1,2]. Moreover, as of 2019, the annual output of oyster shells reached 5.23 million tons [3]. RM is rich in active Al_2_O_3_ and SiO_2_, which can be used to prepare cementitious materials after alkali excitation [4]. Furthermore, the Na_2_O components in RM make the mud alkali-activated [5]. The main component of oyster shell is calcium carbonate. After high-temperature calcination, the content of CaO—an alkaline substance rich in active calcium—can reach more than 90% [6]. Therefore, the preparation of a new type of CPB using IOT, RM, and COS would solve the adverse impact of bulk solid waste on the environment and be beneficial to sustainable development.

Scholars have conducted a number of studies on the material composition and mechanical parameters of CPB. Ercikdi [7] et al. used granular marble waste as additives, with waste bricks applied to partially replace the additives of ordinary Portland cement to prepare a CPB for the backfill of sulfide-rich tailing. The research results showed that the pozzolanic activity of waste bricks could be determined by their particle size. Moreover, marble waste improved the acid buffering capacity of CPB, and CPB samples all met the requirements of strength and durability. Yilmaz [8,9] et al. used construction-industry demolition waste to replace sulfide-rich tailing to prepare CPB. The research results showed that the addition of construction-industry demolition waste can help reduce the porosity of samples and improve compressive strength but has a negative effect on sulfate corrosion. In subsequent studies, the addition of calcium-rich industrial solid waste reduced sulfate formation by up to 72.1%, increased pH of CPB samples, and neutralized sulfide tailing. Liu [10] et al. combined the annealing chaotic competitive neural network to analyze the pores and cracks and proved that the influence of sulfur content on the development of CPB strength is very obvious. Yin [11] et al. found that the increase in solid content is beneficial to the development of CPB strength, but it will deteriorate the working performance of CPB. The solids content to meet CPB strength and workability requirements is 78%. Qiu et al. [12,13] studied the effects of water-film thickness, filler density, and other factors on the fluidity, mechanical strength, ultrasonic waves, and microstructures of CPB. Chen [14] et al. found that the addition of anionic polyacrylamide (APAM) amine to CPB, due to the inhibitory effect of APAM water, led to a decrease in the working performance of CPB and a decrease in the number of hydration products, but inhibited the leaching of Ag and As. Zhang [15] et al. found that temperature has an important effect on the rheological properties of CPB, while pH value has a relatively small effect. Zhao [16] et al. applied acoustic emission technology to the damage detection of tantalum–niobium tailing CPB, and judged the damage according to the fractal characteristics. The research results provided theoretical support for controlling the stability of surrounding stones during backfill.

In recent years, the preparation of CPB from alkali-activated cementitious materials has gradually become a research hotspot. Zhang [17] et al. used alkali-activated slag as a cementitious material and waste rock (mainly containing calcite and dolomite) as an aggregate to prepare alkali-activated paste backfill (APB) and studied the influence of the ratio of sodium oxide to sodium silicate on APB. Jiang [18] et al. used alkali-activated slag to replace cement as the cementitious material to prepare CPB. The authors then studied the impact of solid content, cementitious material content, activator content, alkaline activator mix ratio, and temperature effects on the working properties and compressive strength of CPB samples. Sun [19] et al. used sodium silicate to activate fly ash, cement, and slag as cementitious materials, and coal gangue as aggregate to prepare CPB. The authors also analyzed the impact of the solid mass fraction, fly ash content, and fine ratio effects on the strength and workability of CPB. Chen [20] et al. used Na_2_SO_4_ and CaO to activate copper slag for unclassified lead–zinc mine-tailing backfill, and found that sodium sulfate is related to the formation of calcium silicate hydrogel, and the addition of sodium sulfate makes the internal connection of CPB more compact. The authors also observed chloride deposition in the surface layer, which may lead to a decline in mechanical properties. Cihangir [21] et al. used alkali-activated slag as cementitious material and sulfur-containing tailing as aggregate to prepare CPB and studied the influence of alkali-activated type, concentration, and slag parameters. The results showed that sodium silicate facilitates the formation of early strength and that sodium hydroxide is more beneficial to the development of long-term strength.

Although the preparation of CPB from alkali-activated materials has been extensively studied, the preparation of CPB using COS as an alkaline activator has rarely been reported.

In this study, a new type of CPB was prepared using COS as an alkaline activator, with partial replacement of the slag by RM as the cementitious material and IOT as an aggregate. The CCD method in the response surface was used to design the experiment; the 28-dUCS, slump, and cost of CPB were predicted; and a regression model was established. The mathematical model was optimized using the QGA, and the optimal mix ratio of CPB was determined. Moreover, XRD, FTIR, and SEM-EDS microscopic analyses were carried out on the CPB samples of different ages with the optimal mix ratios.

## 2. Materials and methods

### 2.1. Materials

The raw materials used in this research included IOT, COS, slag, RM, and mixing water.

**Figure 1:**
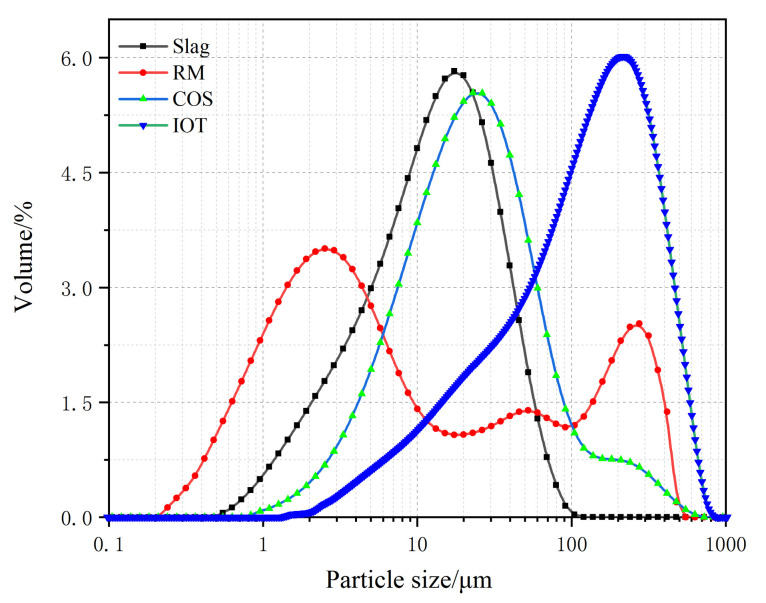
Raw material particle size curve.

**Figure 2:**
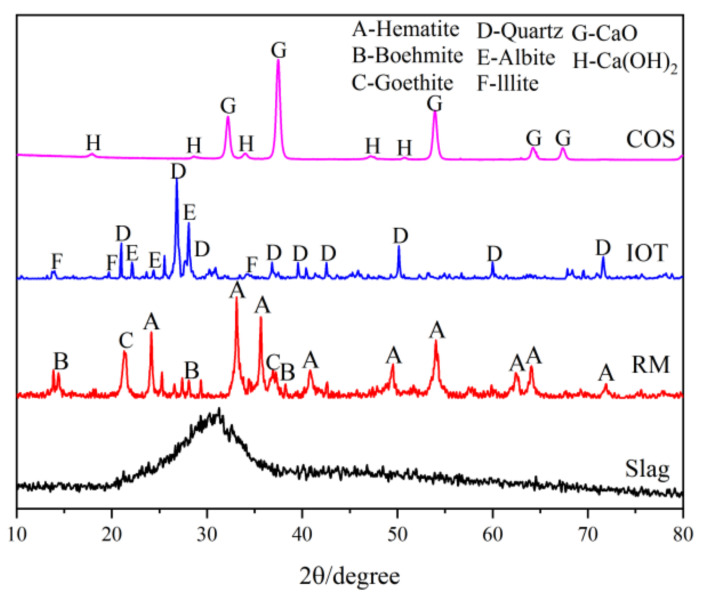
XRD of raw materials (hematite: PDF 01-089-0597; boehmite: PDF 01-072-0359; goethite: PDF 01-081-0464; quartz: PDF 00-046-1045; albite: PDF 00-009-0466; illite: PDF 01-070-3754; CaO: PDF 01-078-0649; and Ca(OH)_2_: PDF 00-004-0733).

(1) IOT: The IOT were obtained from the Baoshan Iron Mine Concentrator (Anshan, China), and dried at 100 °C for 24 h. Table 1 shows the oxide composition analyses (XRF) test results of IOT. Figure 1 shows the particle size curve of IOT. Figure 2 shows the mineral composition of IOT. The specific gravity is 1.79 g/cm^3^.
(2) COS: The oyster shells were taken from the Bohai Sea, Jinzhou City, Liaoning Province, China. The surface attachments were cleaned with a brush, soaked in clean water for 7 days to remove the surface salt, and calcined at 1000 °C for 3 h [6]. Table 1 shows the XRF test results of COS. Figure 1 shows the particle size curve of COS. Figure 2 shows the mineral composition of COS. The specific gravity is 2.25 g/cm^3^.
(3) Slag: The slag is S95 slag produced by Kangjing New Material Technology Co., Ltd. (Jinan, China). Table 1 shows the XRF test results of slag. Figure 1 shows the particle size curve of slag. Figure 2 shows the mineral composition of slag. The slag is mainly amorphous, indicating that the slag was highly active during the polymerization reaction [22]. The specific gravity is 2.91 g/cm^3^.
(4) RM: The RM was taken from the Alumina Factory, Binzhou, China. The RM was dried at 100 °C for 24 h and then mechanically pulverized to ensure uniformity. Table 1 shows the XRF test results of RM. Figure 1 shows the particle size curve of RM. Figure 2 shows the mineral composition of RM. The specific gravity is 2.72 g/cm^3^.
(5) Mixing water: Tap water.

### 2.2. Mix Design

The CCD design method is a more practical design method than the response surface method. It combines mathematical techniques and statistics to establish a function model between the response value and the factor and then carries out the optimization process. The range of the mix ratio was determined according to the previous test, and the mix ratio was designed using the CCD method. Table 2 shows the mix ratio data of the response surface designed by CCD.

### 2.3. Experimental Methods 

**Figure 3:**
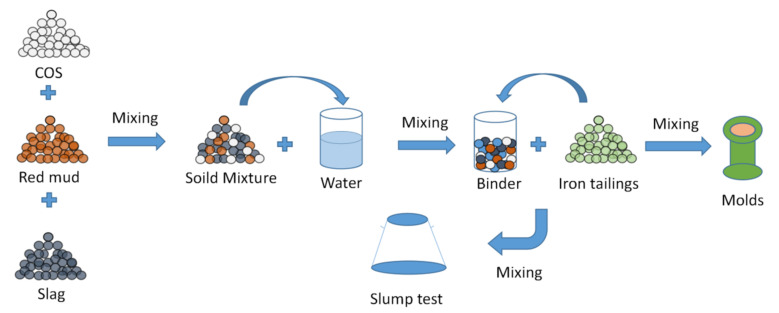
Preparation process for the samples.

First, the COS, slag, and RM were mixed and stirred for 3 min. Then, water was poured into the solid mixture and stirred for 3 min. The IOT was poured into the binder and stirred again for 3 min [23]. Finally, a slump test was carried out and the sample was put into a Φ 50 × 100 mm^2^ cylinder-shaped mold. Then, the sample was put into a SHBY-90B curing box (temperature: 20 ± 5 °C, humidity: greater than 96%) for curing [23]. Figure 3 provides a flow chart illustrating the preparation of the CPB samples.

The compressive strength of CPB was tested according to the specifications in [24]. The sample is loaded via displacement with a strain rate of 1 mm/min until the sample failed. During the loading process, the axial deformation and stress are recorded by the data acquisition system. The slump was tested according to the specifications in [25]. CPB sample densities were tested according to [26] and costed according to the material cost values presented in Table 3.

SEM was used to observe the morphology of the hydration products (under 5000× conditions) using a TescanMira4 scanning electron microscope and EDS analysis was performed at the same time. FTIR spectra were tested with a Thermo Scientific Nicolet iS20 (wavenumbers range 400–400 cm^−1^). The mineral composition of the samples was analyzed by XRD using a SmartLab-SE device with Cu Kα radiation, with scanning in the range of 10–80° 2θ.

## 3. Results 

### 3.1. CCD Method Test Results 

Table A1 (in Appendix A) shows specific mix ratios and response values. Factor 1 is the COS content (code A), factor 2 is the RM substitution rate (code B), factor 3 is the solid mass (code C), and factor 4 is the aggregate–binder ratio (code D). Response 1 is 28-dUCS (MPa), response 2 is slump (mm), and response 3 is cost (USD/m^3^). Combined with the mix ratio and test results in Table A1, a second-order model was used for fitting [27]. Then, a regression model is established using the four factors A, B, C, and D alongside CPB 28-dUCS, slump, and cost, as outlined in Equations (1)–(3):

*R*_1_ (28-dUCS) fitting equation:

*R*_1_ = −6312.8 + 0.93*A* − 2.61*B* + 154.15*C* − 28.93*D* + 8.25 × 10^− 3^*AB* − 6.25 × 10^− 3^*AC* +0.09*AD* + 0.03*BC* − 0.01*BD* + 0.43*CD* − 0.03*A*^2^ − 1.79 × 10^− 4^*B*^2^ − 0.94*C*^2^ − *D*^2^ (1)

*R*_2_ (slump) fitting equation:

*R*_2_ = −88668.05 + 188.34*A* − 96.67*B* + 2268.77*C* − 1317.46*D* + 0.135*AB* − 2.38*AC* +1.45*AD* + 1.11*BC* + 2.13*BD* + 17.38*CD* − 0.24*A*^2^ − 0.16*B*^2^ − 14.47 *C*^2^ − 16.38*D*^2^ (2)

*R*_3_ (cost) fitting equation:

*R*_3_ = −3.59 − 0.08*A* − 0.03*B* + 0.3*C* − 1.56*D* + 5 × 10^−5^
*AB* + 1.25 × 10^−3^
*AC* − 2.5 × 10^−3^
*AD* −6.25 × 10^−4^
*BC* + 8.25 × 10^−3^
*BD* − 3.75 × 10^−3^
*CD* − 2.5 × 10^−4^
*A*^2^ − 6.25 × 10^−5^
*B*^2^ − 1.56 × 10^−3^
*C*^2^ + 0.12*D*^2^ (3)

### 3.2. Analysis of Variance 

**Figure 4:**
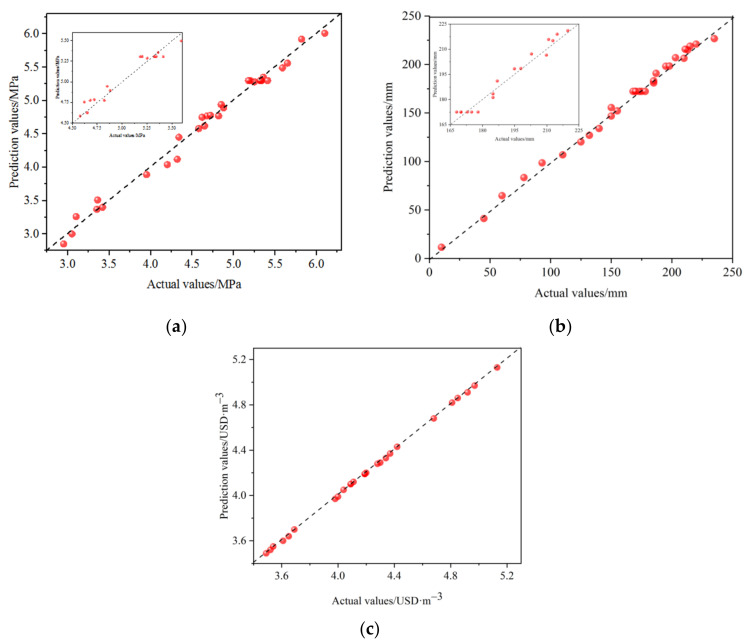
Prediction value and actual value for (**a**) 28-dUCS, (**b**) slump, and (**c**) cost.

The analysis of variance data for 28-dUCS, slump, and cost are shown in Table A2. Figure 4 shows the relationship between the actual and prediction values of 28-dUCS, slump, and cost.

**Figure 5:**
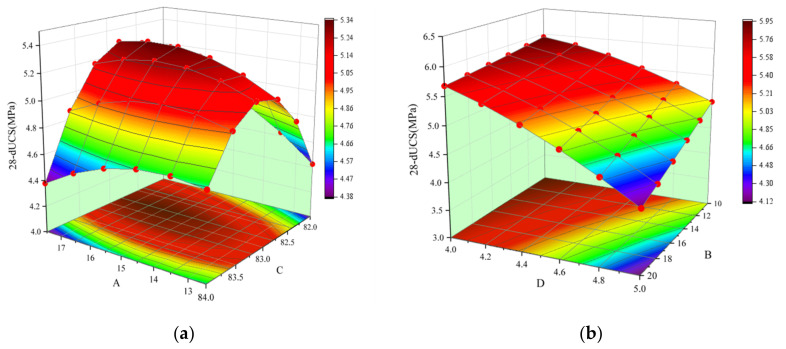
The interaction of *AC* and *BD* under 28-dUCS: (**a**) The effect of *AC* under 28-dUCS and (**b**) the effect of *BD* under 28-dUCS.

According to the data in Table A2 (in Appendix B), the *p*-value of the 28-dUCS regression model is less than 0.0001, which shows that the model is very significant. In addition, the missing fitting term *p* = 0.1391 (greater than 0.05) and the variance of 0.9887 both indicate that the regression model fits well. The influence of factors *A*, *B*, *C*, and *D* for 28-dUCS is very significant; the order of influence is *D* > *B* > *C* > *A*. In the interaction, the *p*-values of the *AB*, *AC*, and BD items are all less than 0.05, and the effect for 28-dUCS is significant. As an example, Figure 5 shows a 3D map of the response surface of the interaction between *AC* and *BD*. In Figure 5a, with an increase in *A* and *C*, the 28-dUCS response surface curvature (intensity change rate) presents a trend of first decreasing and then increasing. The 28-dUCS response surface curvature (intensity growth rate) in Figure 5b shows a decreasing trend with a decrease in *B* and *D*. The order of interaction between different factors for 28-dUCS is *AC* > *BD* > *AB* > *CD* > *AD* > *BC*.

**Figure 6:**
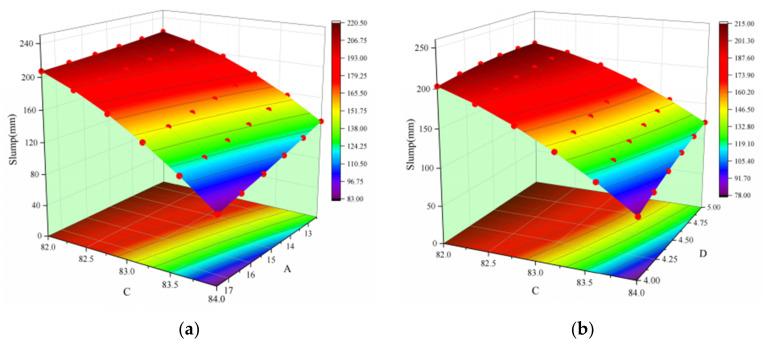
The effect of *AC* and *CD* interactions for slump: (**a**) the effect of *AC* for slump and (**b**) the effect of *CD* for slump.

According to the data in Table A2, the *p*-value of the slump regression model is less than 0.0001, which shows that the model is very significant. In addition, the missing fitting term *p* = 0.1128 (greater than 0.05) and the variance of 0.9945 both indicate that the regression model fits well. The influence of factors *A*, *B*, *C*, and *D* for slump was very significant, and the order of influence was *C* > *A* > *D* > *B*. In the interaction, the *p*-values of *AB*, *AC*, *BC*, *BD*, and *CD* are all less than 0.05, indicating that the effect for slump was significant. As an example, Figure 6 shows a 3D map of the response surface for the interaction of *AC* and *CD*. In Figure 6a, the slump response surface curvature (slump growth rate) shows a decreasing trend as *A* and *C* decrease. In Figure 6b, with *C* increases and *D* decreases, the slump response surface curvature shows a decreasing trend. The order of the interactions between the different factors for slump is *CD* > *AC* > *BC* > *AB* > *BD* > *AD*.

**Figure 7:**
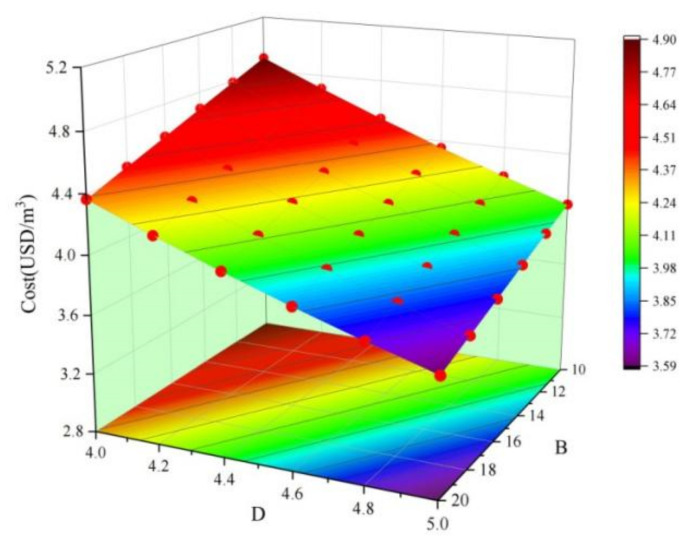
The effect of *BD* interactions for cost.

According to the data in Table A2, the *p*-value of the cost regression model is less than 0.0001, which shows that the model is very significant. The variance is 0.9998, indicating that the regression model fits well. The influence of factors *A*, *B*, *C*, and *D* on cost is very significant, and the order of influence is *D* > *B* > *C* > *A*. In the interaction, the *p*-value of the *BD* term is found to be less than 0.05, and the effect on cost is significant. As an example, Figure 7 shows a 3D map of the response surface of the *BD* interaction. When *B* and *D* decrease, the cost response surface curvature (cost value growth rate) decreases. The order of interaction between the different factors for cost is *BD* > *AC* > *AD* > *BC* = *CD* > *AB*.

### 3.3. Quantum Genetic Algorithm Multi-Objective Optimization

To determine the optimal mix ratio of CPB, we used the quantum genetic algorithm to solve the CPB parameter optimization problem. Here, the fitness function is established by using the regression model created via the CCD method, as shown in Formula (4):

(4) $$Y=\left\{\begin{array}{ll} 0 & \left(R_{2}<200\;\mathrm{mm}\right)\\ \frac{R_{1}}{R_{3}} & \left(R_{2}\geq 200\;\mathrm{mm}\right) \end{array}\right.$$

where *R*_1_ represents the 28-dUCS regression model, *R*_2_ represents the slump regression model, and *R*_3_ represents the cost regression model. The value range of the independent variable is $A\in \left[12.5,17.5\right]$, $B\in \left[10,20],\;C\in [82,84\right]$, and $D\in \left[4,5\right]$. The fitness function looks for two mutually restrictive performance indicators in the state where slump is not less than 200 mm. Then, cost is minimized, and 28-dUCS is maximized. The larger the value is, the higher the fitness will be, indicating that the optimization effect is better.

The quantum genetic algorithm works as follows:

(1) Initialize the population of chromosomes, defined by [28]:
  (5) $$c_{i}=\left[c_{i1},c_{i2},\cdots ,c_{kd}\right]$$
  (6) $$\left\{\begin{array}{c} c_{ij}={\left[\alpha_{ij},\beta_{ij}\right]}^{T},j=1,2,\cdots ,d\\ \alpha_{ij}={\left[\alpha_{ij}^{1},\alpha_{ij}^{2},\cdots ,\alpha_{ij}^{k}\right]}^{T}\\ \beta_{ij}={\left[\beta_{ij}^{1},\beta_{ij}^{2},\cdots ,\beta_{ij}^{k}\right]}^{T} \end{array}\right.$$
  where $c_{ij}$ represents the j dimension element of $c_{i}$. The values of ${\left|\alpha_{ij}^{l}\right|}^{2}$ and ${\left|\beta_{ij}^{l}\right|}^{2}$ represent the probability of each Q-bit appearing in a 0 or 1 state.
(2) Calculate the fitness value according to Equation (4) and assign $c_{i}$. Through all independently observed Q-bits, search for the chromosome that satisfies the highest fitness. The optimal individual can be gradually observed and then retained. Then, quantum crossover and mutation operations are carried out through Equation (7) to produce excellent individuals [28]:
  (7) $$c_{mj}=\left(\begin{array}{c} \alpha_{mj}^{1},\cdots ,\alpha_{nj}^{l},\cdots ,\alpha_{mj}^{k}\\ \beta_{mj}^{1},\cdots ,\beta_{nj}^{l},\cdots ,\beta_{mj}^{k} \end{array}\right)\to c_{nj}=\left(\begin{array}{c} \alpha_{nj}^{1},\cdots ,\alpha_{mj}^{l},\cdots ,\alpha_{nj}^{k}\\ \beta_{nj}^{1},\cdots ,\beta_{mj}^{l},\cdots ,\beta_{nj}^{k} \end{array}\right)$$
  where $c_{mj}$ and $c_{nj}$ are the j dimension elements of the two selected chromosomes, $c_{m}$ and $c_{n}$, respectively.
(3) Quantum gate update: The update process is as shown in Equation (8) [28]:
  (8) $$\left\{\begin{array}{c} G_{ij}^{l}\left(\theta \right)=\left[\begin{array}{cc} \cos\theta_{ij}^{l} & -\sin\theta_{ij}^{l}\\ \sin\theta_{ij}^{l} & \cos\theta_{ij}^{l} \end{array}\right]\\ \theta_{ij}^{l}=sg\left(\alpha_{ij}^{l},\beta_{ij}^{l}\right)∆\theta_{ij}^{l} \end{array}\right.$$
  where $\theta_{ij}^{l}$ is the l rotation angle applied to $c_{i}$, $∆\theta_{ij}^{l}$ is the step factor, $sg\left(\alpha_{ij}^{l},\beta_{ij}^{l}\right)$ is the symbolic function, and $g_{hj}^{l}$ represents the lth bit of the hth niche center where $c_{i}$ belongs. The updated chromosomes are then evaluated and compared with the parents, and only the best individuals are retained.
(4) If the algorithm reaches the pre-defined maximum number of iterations $I_{max}$ (in this study, $I_{max}=300$), the algorithm stops the search; otherwise, the algorithm returns to step (2) to continue the search.

**Figure 8:**
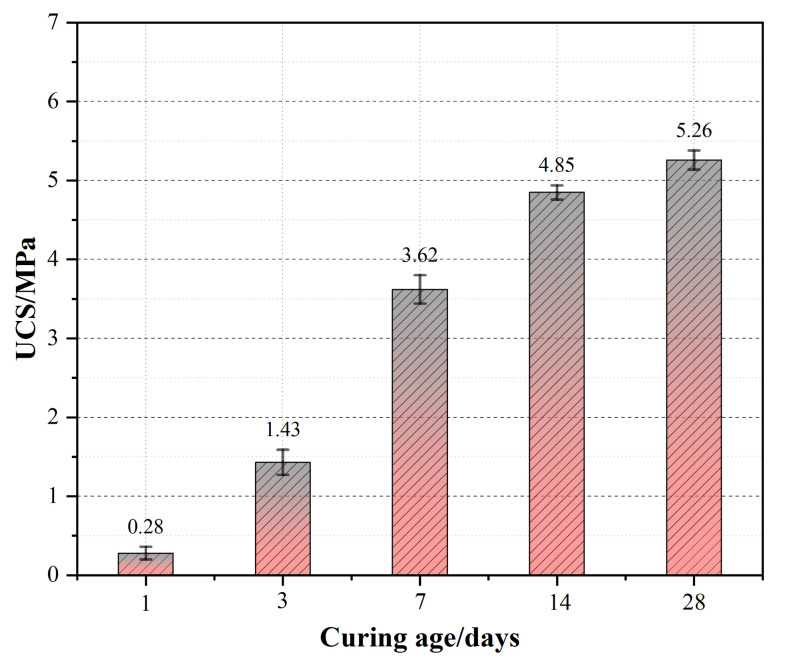
Compressive strength of CPB samples with the optimal mix ratios at different ages.

After optimizing the quantum genetic algorithm, the maximum fitness is found to be 1.265. At this time, the COS dosage was 16.87%, the RM substitution rate was 17.50%, the solid mass was 82.24%, and the aggregate–binder ratio was 4.37. Table 4 shows data comparing the predicted and experimental values. Figure 8 shows the compressive strength of CPB samples with the optimal mix ratio at different ages.

### 3.4. Microscopic Analysis

#### 3.4.1. XRD

**Figure 9:**
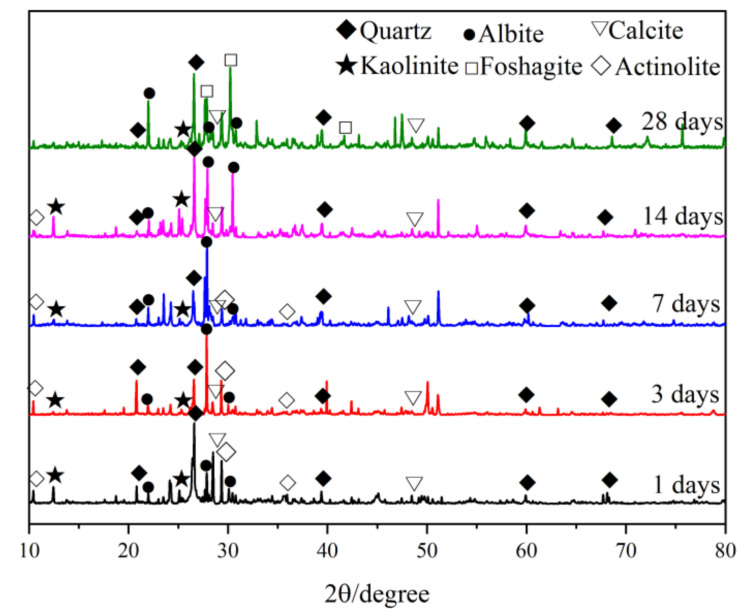
XRD of CPB samples with the optimal mix ratios at different ages (quartz: PDF 00-046-1045; kaolinite: PDF 01-078-2110; albite: PDF 00-009-0466; foshagite: PDF 01-074-0360; calcite: PDF 01-083-0577; and actinolite: PDF 01-085-2157).

To determine the phase composition of the CPB samples of different ages with the optimal mix ratio, XRD tests are carried out. Figure 9 shows the XRD analysis results for the CPB samples at different ages. Table 5 shows the mineral content of the CPB samples at different ages. As expected, no lime in COS is found in samples of any age, indicating that the lime in COS participated in the polymerization reaction, providing an alkaline environment for the CPB system.

At an age of 1 d, the content of quartz (SiO_2_) was 34.4%, which was mainly from IOT, and the content of albite (NaAlSi_3_O_8_) was 18.3%, which is also the main mineral in the IOT. The content of kaolinite (Al_4_(OH)_8_Si_4_O_10_) was 17.2%, and kaolinite is not found in the XRD results of the raw material analysis, indicating that kaolinite was an early polymerization product. Actinite (Na_0.15_K_0.04_Ca_1.68_(Fe_1.42_Mg_3.68_)Si_7.38_Al_0.83_O_22_(OH)_2_) content was 22.3%. For the chemical composition of actinite, the iron content was 9.3% and no hematite or goethite in RM were found in the XRD analysis results, indicating that the hematite and goethite in RM were dissolved in the alkaline medium [4]. The content of calcite (CaCO_3_) is 7.8%. This calcite was a decryption product of the C–S–H and C–A–S–H amorphous gel [6], which may be produced by the carbonization of calcium hydroxide.

With an increase in curing age, amorphous parts of the crystals in the CPB system were gradually dissolved in the alkaline medium. According to the results of crystal content at 3 and 7 d of age, the quartz content was 17.8% and 12.7%, respectively, showing an obvious decreasing trend. This result indicates that the quartz in IOT dissolved in the alkaline medium. The albite content was 30.8% and 35%, and the actinite content was 29.1% and 38.3%, indicating that albite and actinite were polymerization products. The percentage of kaolinite, another early polymerization product, showed a decreasing trend. The content of calcite also gradually increased.

At the age of 14 d, we found that the quartz content was 32.5%, while the contents of albite and actinite were 34% and 17.7%, possibly due to the gradual transformation of albite and actinite into more stable quartz. The kaolinite content was 3.9%, and the calcite content was 11.9%, which maintained a decreasing and increasing trend, respectively.

At the age of 28 d, we found foshagite (Ca_4_(Si_3_O_9_)(OH)_2_) in the range of 26–33° at 2θ. Moreover, the percentage of quartz and albite decreased, and actinite disappeared, indicating that this mineral component was converted into more stable foshagite.

#### 3.4.2. FTIR

**Figure 10:**
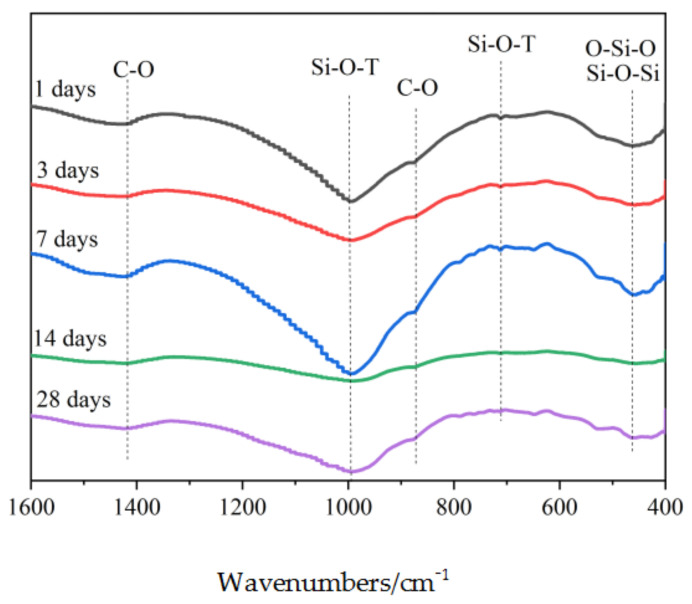
FTIR of CPB samples with the optimal mix ratios at different ages.

Since the XRD patterns cannot distinguish between the C–S–H and C–A–S–H amorphous gels, in order to further determine the phase combinations of the CPB samples, FTIR tests are performed on the CPB samples of different ages. Figure 10 shows the results of the FTIR spectral analysis of CPB samples.

Combined with the previous research results [29,30], we observed a sharp peak at 450 cm^−1^ for Si–O–Si and O–Si–O bending vibrations and a peak at 715 cm^−1^ attributed to the CPB sample Si–O–T (symmetric stretching vibrations in middle aluminosilicate composites). The C–O bending vibration at 870 cm^−1^ and the C–O asymmetric stretching vibration at 1470 cm^−1^ were observed to be the peak characteristics in calcite. Here, the peak near 966 cm^−1^ is the Si–O–T asymmetric stretching vibration in C–A–S–H.

**Figure 11:**
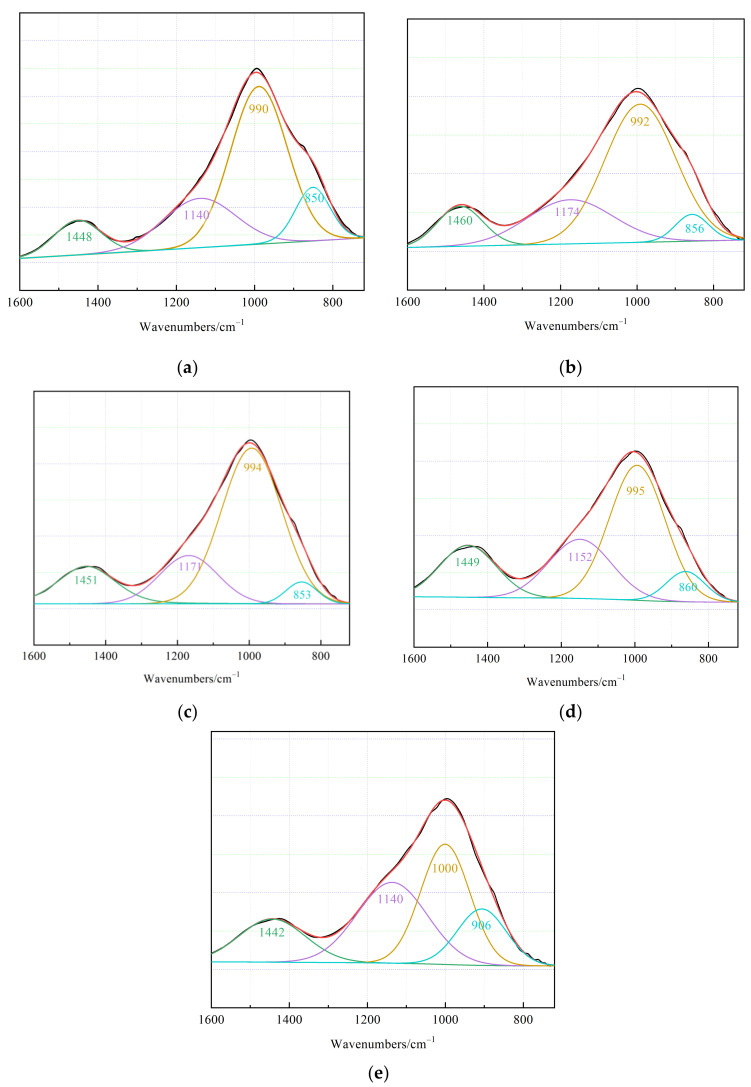
Deconvolution diagram of asymmetric stretching vibrations for the Si–O–T bonds of C–S–H and C–A–S–H at different CPB sample ages with the optimal mix ratios: (**a**) 1 day, (**b**) 3 days, (**c**) 7 days, (**d**) 14 days, and (**e**) 28 days.

Because Si–O–T mainly vibrates in the region of 800–1300 cm^−1^ [29], for a clearer representation, the deconvolution results are shown in Figure 11. The peak (Q^2^) located near 1140 cm^−1^ is Si–O–T in the C–S–H stretching vibration peaks. Except for the 1-day age, curing age was from 3 days to 28 days, and the (Q^1^) C–O stretching vibration peak and Q^2^ peak located near 1470 cm^−1^ gradually shifted to a lower wavenumbers direction, indicating that the polymerization degree of carbonated C–S–H increased [6]. At an age of 1 day, a high degree of polymerization was observed because highly polymerized silicate materials in the precursor had not been fully dissolved, which is consistent with the analysis results of XRD.

However, we found that the Si–O–T peak (Q^3^) near 990 cm^−1^ and the C–O stretching vibrational peak (Q^4^) near 870 cm^−1^ shifted to higher wavenumbers, indicating a decreasing degree of polymerization for the carbonated C–A–S–H. The degree of polymerization of the two gels presented different trends. Because the bond energy of Al–O is lower than that of Si–O [30], Al entered the amorphous material and participated in polymerization at the beginning of the reaction. Thus, the degree of aggregation was the highest at an age of 1 day. In a previous analysis [6], the degree of C–A–S–H polymerization is determined by Al/Si; when Al/Si increased, the peak shifted in the direction of a high wavenumbers, and the degree of polymerization decreased. A decrease in Al/Si in C–A–S–H may also indicate the conversion of C–A–S–H to C–S–H.

#### 3.4.3. SEM-EDS 

**Figure 12:**
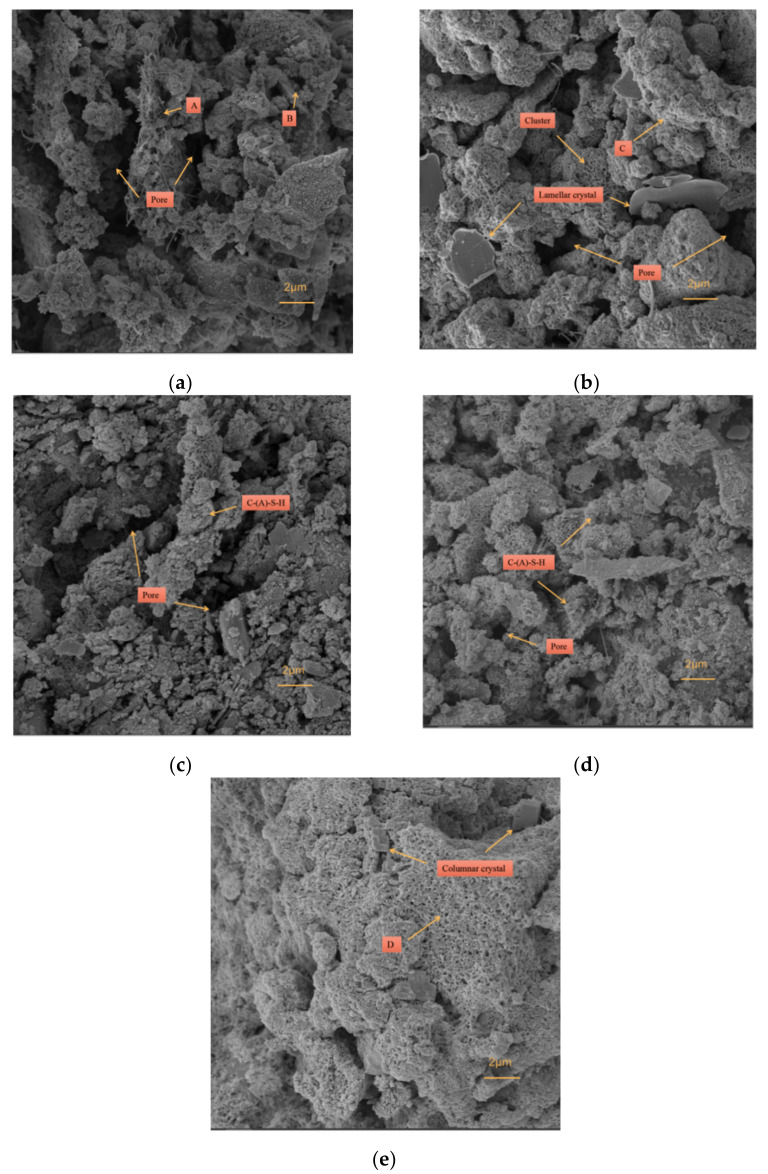
SEM results of CPB samples with the optimal mix ratios at different ages: (**a**) 1 day, (**b**) 3 days, (**c**) 7 days, (**d**) 14 days, and (**e**) 28 days.

**Figure 13:**
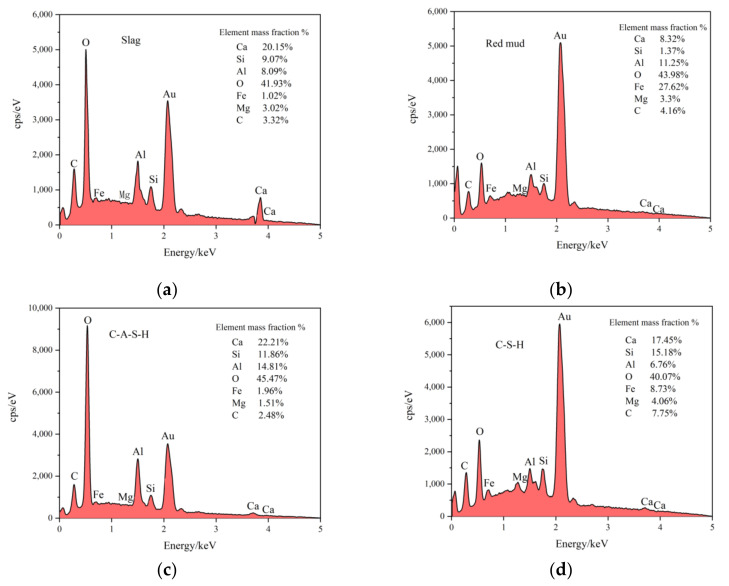
EDS analysis results: (**a**) point A, (**b**) point B, (**c**) point C, and (**d**) point D.

To further illustrate the internal morphology of the CPB samples of different ages with the optimal mix ratios, SEM-EDS tests were carried out. Figure 12 shows the microstructures changes of the CPB samples with optimal mix ratios at different ages (imaging from SEM), and Figure 13 shows the EDS analysis data.

Figure 12a show the microstructures of the one-day-old CPB sample, with irregular plate-like substances intertwined with C–S–H and C–A–S–H filling the internal pores. These substances are the main source of early compressive strength in the CPB samples. In addition, a large amount of irregular needle-like material is visible at point A, which was found to be dissolved slag based on the EDS analysis in Figure 13a [31]. Due to the insufficient dissolution of the slag and the low content of polymerized products, the internal pores of the CPB sample were poorly filled. Figure 12a shows that large-sized pores are detrimental to compressive strength. We also observed particulate matter with smaller particle sizes at point B. Combined with the EDS analysis in Figure 13b, the iron content was found to be as high as 27.62%, indicating that the material was undissolved red mud.

Figure 12b,c show the microstructures of the three-day-old and seven-day-old CPB samples, respectively. The irregular rod-like substances gradually transformed into clusters and finally disappeared. Additionally, large-area C–S–H and C–A–S–H gel and large-sized flaky crystals appeared. The results indicated that under alkaline conditions, with the dissolution of the precursor material, the content of amorphous gel and crystals increased, and the pore structure inside the CPB was significantly improved. EDS analysis was also carried out at point C. Here, the iron content was found to be 1.96%, indicating that with an increase in curing age, RM gradually dissolved. Moreover, the contents of aluminum and silicon were 14.81% and 11.86%, respectively. We determined that the material contained a small amount of iron from the C–A–S–H gel. This result is consistent with the XRD analysis results.

Figure 12d shows the microstructures of the CPB sample at 14 d. Here, the internal structure of the CPB sample was tightly connected by C–S–H and C–A–S–H. Although some pores are visible, their number is significantly reduced. Furthermore, the degree of crystallinity increased and the particle size became more refined. Additionally, the compaction of the samples increased, significantly improving compressive strength [32].

Figure 12e shows the microstructures of the CPB sample at 28 d, illustrating the appearance of columnar crystals filling the pores. Combined with XRD analysis, the columnar substances were silicate or aluminosilicate crystals with a more stable state and higher crystallinity. The EDS analysis of point D shows that the iron content was 8.73%, indicating that with an increase in curing age, the RM dissolved more fully. Here, the aluminum and silicon contents were 6.76% and 15.18%, respectively. We determined that the material contained a large amount of iron from the C–S–H gel. The internal pores of the CPB samples were fully filled with C–S–H, C–A–S–H, and crystals; the connections are more compact; and the compressive strength reached the maximum value at all ages, indicating that aluminosilicate hydrates determined the compressive strength of the CPB samples [31].

## 4. Conclusions

(1) In this study, a new type of red mud, COS, slag, and tailing composite cemented paste backfill was developed. When the COS content is 16.87%, the RM substitution rate is 17.50%, the solid mass is 82.24%, and the aggregate–binder ratio was 4.37, which represents the optimal mix ratio.
(2) With an increase in curing age, the quartz in the IOT and the hematite goethite in RM gradually dissolved and participated in the polymerization reaction, and the quartz content increased at an age of 14 days. The appearance of foshagite at an age of 28 days indicated the transformation of aluminosilicate crystals to a more stable state.
(3) The higher degree of C–S–H polymerization observed at 1 d of age was caused by the insufficient dissolution of silicate substances. With an increase in curing age, the degree of polymerization of C–S–H in the amorphous gel gradually increased, and the degree of polymerization of C–A–S–H gradually decreased.
(4) The precursor material gradually dissolved, and the content of C–S–H and C–A–S–H amorphous gel and aluminosilicate crystals increased, which effectively filled the pores inside the CPB, thereby improving compressive strength.

## Figures and Tables

**Table 1:** Chemical composition of COS, RM, slag, and IOT determined by XRF (wt%).

Materials	CaO	SiO_2_	Al_2_O_3_	MgO	Fe_2_O_3_	SO_3_	Na_2_O	TiO_2_	LOI	Others
COS	95.71	0.21	0.06	0.29	0.05	0.75	0.99	--	1.10	0.84
RM	2.52	8.99	18.50	0.15	48.24	0.33	8.18	8.18	3.50	1.41
Slag	33.13	30.21	19.38	10.19	0.21	2.03	0.52	0.97	2.90	0.46
IOT	5.68	40.87	13.16	4.82	17.00	0.54	3.98	--	11.9	2.05

**Table 2:** CCD design scheme.

Factor	Code	Unit	Level				
			−2	−1	0	1	2
COS dosage	A	%	10	12.5	15	17.5	20
RM substitution rate	B	%	5	10	15	20	25
Solid mass	C	%	81	82	83	84	85
Aggregate-binder ratio	D	-	3.5	4.0	4.5	5.0	5.5

**Table 3:** Material Cost Values.

Materials	COS	Slag	Water
Unit price (USD/kg)	0.0047	0.0137	0.000665

**Table 4:** Comparison of predicted values and actual values of the CPB samples with the optimal mix ratios.

Term	28-dUCS (MPa)	Slump (mm)	Cost (USD/m^3^)
Experimental	5.26	202	4.16
Predicted	5.24	200	4.15

**Table 5:** Contents of mineral components in CPB samples with the optimal mix ratios at different ages (wt%).

	1 Days	3 Days	7 Days	14 Days	28 Days
Quartz	34.4	17.8	12.7	32.5	23.6
Albite	18.3	30.8	35.0	34.0	33.2
Calcite	7.8	9.0	10.2	11.9	17.6
Kaolinite	17.2	13.3	3.9	3.9	2.1
Actinolite	22.3	29.1	38.3	17.7	--
Foshagite	--	--	--	--	23.6

## Data Availability

Date can be obtained from corresponding authors upon reasonable request.

## Appendix A

**Table A1:** Test results of the CCD method.

Test Number	Factor				Response		
	1	2	3	4	1	2	3
	*A* (%)	*B* (%)	*C* (%)	*D*	28-dUCS (MPa)	Slump (mm)	Cost (USD/m^3^)
1	15	15	83	4.5	5.18	170	4.19
2	12.5	20	82	5	3.05	220	3.52
3	15	25	83	4.5	4.82	185	3.69
4	12.5	10	82	4	5.25	213	4.81
5	17.5	10	82	5	4.58	198	4.00
6	12.5	10	82	5	4.32	211	3.98
7	17.5	10	82	4	5.65	187	4.85
8	15	15	83	4.5	5.41	168	4.19
9	15	15	85	4.5	2.95	10	4.28
10	15	5	83	4.5	5.82	155	4.68
11	15	15	83	3.5	6.1	132	5.13
12	15	15	83	5.5	3.36	185	3.49
13	12.5	10	84	4	5.36	93	4.92
14	15	15	83	4.5	5.33	175	4.19
15	10	15	83	4.5	4.65	210	4.11
16	17.5	10	84	4	4.72	45	4.97
17	12.5	20	84	5	3.35	150	3.61
18	17.5	20	82	4	5.59	195	4.34
19	15	15	81	4.5	3.1	235	4.09
20	15	15	83	4.5	5.32	173	4.19
21	17.5	10	84	5	4.2	78	4.09
22	17.5	20	84	4	4.62	60	4.42
23	15	15	83	4.5	5.34	178	4.19
24	12.5	20	82	4	4.68	203	4.30
25	20	15	83	4.5	4.85	150	4.20
26	12.5	20	84	4	4.88	110	4.37
27	15	15	83	4.5	5.2	170	4.19
28	17.5	20	84	5	3.42	125	3.65
29	12.5	10	84	5	4.34	140	4.04
30	17.5	20	82	5	3.95	215	3.54

## Appendix B

**Table A2:** Analysis of variance table.

Source	Sum of Squares			Mean Square			F Value			*p*-Value		
	*R*_1_	*R*_2_	*R*_3_	*R*_1_	*R*_2_	*R*_3_	*R*_1_	*R*_2_	*R*_3_	*R*_1_	*R*_2_	*R*_3_
Model	22.26	89,089.88	5.57	1.59	6363.56	0.4	93.72	192.03	4394.48	<0.0001	<0.0001	<0.0001
*A*	0.15	5310.38	0.01	0.15	5310.38	0.01	8.86	160.25	110.48	0.0094	<0.0001	<0.0001
*B*	1.97	1247.04	1.45	1.97	1247.04	1.45	116.24	37.63	15,962.62	<0.0001	<0.0001	<0.0001
*C*	0.26	69,445.04	0.051	0.26	69,445.04	0.051	15.1	2095.58	566.92	0.0015	<0.0001	<0.0001
*D*	9.4	4732.04	4.03	9.4	4732.04	4.03	553.99	142.79	44,461.15	<0.0001	<0.0001	<0.0001
*AB*	0.19	232.56	6.25 × 10^−6^	0.19	232.56	6.25 × 10^−6^	11.15	7.02	0.069	0.0045	0.0182	0.7963
*AC*	0.74	1105.56	3.063 × 10^−4^	0.74	1105.56	3.063 × 10^−4^	43.59	33.36	3.38	<0.0001	<0.0001	0.0858
*AD*	0.029	45.56	1.562 × 10^−4^	0.029	45.56	1.562 × 10^−4^	1.7	1.37	1.73	0.2115	0.2593	0.2087
*BC*	2.03 × 10^−3^	264.06	5.625 × 10^−5^	2.03 × 10^−3^	264.06	5.625 × 10^−5^	0.12	7.97	0.62	0.7345	0.0129	0.4429
*BD*	0.38	175.56	6.806 × 10^−3^	0.38	175.56	6.806 × 10^−3^	22.29	5.3	75.16	0.0003	0.0361	<0.0001
*CD*	0.063	1207.56	5.625 × 10^−5^	0.063	1207.56	5.625 × 10^−5^	3.68	36.44	0.62	0.0742	<0.0001	0.4429
*A* ^2^	0.46	31.57	1.765 × 10^−3^	0.46	31.57	1.765 × 10^−3^	27.23	0.95	19.49	0.0001	0.3445	0.0005
*B* ^2^	4.43 × 10^−3^	55.86	7.44 × 10^−6^	4.43 × 10^−3^	55.86	7.44 × 10^−6^	0.26	1.69	0.082	0.6168	0.2138	0.7783
*C* ^2^	8.63	4853.36	7.44 × 10^−6^	8.63	4853.36	7.44 × 10^−6^	508.83	146.46	0.082	<0.0001	<0.0001	0.7783
*D* ^2^	0.5	507.65	0.026	0.5	507.65	0.026	29.37	15.32	286.02	<0.0001	0.0014	<0.0001
Residual	0.25	497.08	1.358 × 10^−3^	0.017	497.08							
Lack of fit	0.22	427.75	1.358 × 10^−3^	0.022	427.75	1.358 × 10^−3^	2.74	3.08		0.1391 0.1128		
Pure error	0.039	69.33	0	7.87 × 10^−3^	69.33	0						
Cor total	22.52	89,586.97	5.57		89,586.97							
Std. dev.	0.13	5.76	9.516 × 10^−3^		R-Squared		0.9887	0.9945	0.9998			
Mean	4.65	157.97	4.21		Adj R-squared		0.9781	0.9893	0.9995			
C.V.%	2.8	3.64	0.23		Pred R-squared		0.9424	0.9714	0.9986			
PRESS	1.3	2563.68	7.824 × 10^−3^		Adeq R-squared		34.344	52.859	243.478			
